# Survival from cardiac arrest at a core temperature of 14.0 °C on hospital arrival caused by cold exposure hypothermia treated with extracorporeal cardiopulmonary resuscitation: a case report

**DOI:** 10.1016/j.resplu.2026.101265

**Published:** 2026-02-11

**Authors:** Hitoshi Kano, Masaki Nagama, Keisuke Bando, Akio Endo, Toru Takiguchi, Yutaka Igarashi, Shoji Yokobori

**Affiliations:** a Emergency and Critical Care Center, Sapporo City General Hospital, Kita 11, Nishi 13, Chuo-ku, Sapporo 060-8604, Japan; b Department of Emergency and Critical Care Medicine, Nippon Medical School, 1-1-5 Sendagi, Bunkyo-ku, Tokyo 113-8603, Japan; c Department of Emergency and Critical Care Medicine, University of the Ryukyus, 207 Uehara, Nishihara, Okinawa 903-0215, Japan; d Division of Critical Care, Heiseikai Hospital, Kita 1, Nishi 18, Chuo-ku, Sapporo 060-0001, Hokkaido, Japan

**Keywords:** Cold exposure, Accidental hypothermia, Cardiac arrest, Extracorporeal cardiopulmonary resuscitation, Neuroprotection, HOPE score

## Abstract

**Background:**

Accidental hypothermia complicated by cardiac arrest remains a challenging clinical condition, in which extracorporeal cardiopulmonary resuscitation (ECPR) may provide favorable outcomes in selected patients (Ruttmann et al., 2007; Walpoth et al., 1990).

**Case presentation:**

A 70-year-old woman was found collapsed on a street near her home and was in cardiac arrest when emergency medical services arrived. On hospital arrival, her rectal temperature was 14.0 °C, and serum potassium measured from arterial blood was 8.68 mmol/L. Accidental cold exposure hypothermia was presumed, and ECPR using veno-arterial extracorporeal membrane oxygenation (VA-ECMO) was initiated (Ruttmann et al., 2007; Walpoth et al., 1990). Ventricular fibrillation occurred shortly after initiation of ECPR, and defibrillation was performed when the core body temperature reached 25.6 °C, resulting in return of spontaneous circulation and a favorable neurological outcome. The predicted survival probability according to the HOPE score was 40% (Pasquier et al., 2018).

**Conclusion:**

This case represents survival at one of the lowest core body temperatures reported on hospital arrival in an adult and highlights the established neuroprotective effects of profound hypothermia in accidental hypothermic cardiac arrest (Brown et al., 2012; McCullough and Arora, 2004; Gilbert et al., 2000; Sessler, 1997).

## Highlights

• A patient with accidental hypothermic cardiac arrest arrived with a core temperature of 14.0 °C.
• Extracorporeal cardiopulmonary resuscitation allowed survival with favorable neurological outcome.
• Profound hypothermia may confer cerebral protection despite prolonged circulatory arrest.
• This case highlights the clinical importance of ECPR in carefully chosen hypothermic cardiac arrest patients.

## Background

Extracorporeal cardiopulmonary resuscitation (ECPR) using extracorporeal membrane oxygenation (ECMO) has been established as an effective treatment for accidental hypothermia complicated by cardiac arrest.^1^, ^2^ Accidental hypothermia may occur in various settings, including immersion, avalanche burial, drowning, and cold exposure.^4^, ^5^ Among these etiologies, cold exposure hypothermia is often characterized by a gradual decline in core body temperature and frequently occurs in unwitnessed circumstances.

In normothermic cardiac arrest, prolonged no-flow time (NFT) is strongly associated with poor neurological outcomes.^8^ In contrast, in accidental hypothermia, favorable neurological recovery has been reported even after unwitnessed cardiac arrest, particularly when extracorporeal rewarming is applied.^1^, ^2^, ^4^

## Case report

A 70-year-old woman was found collapsed on a street near her home in Sapporo, Japan. The ambient temperature at the time of discovery was approximately −10 °C.^9^ According to information obtained after admission, the patient had fallen near her home and had been unable to move thereafter. There was no evidence of intoxication, and no comorbidities directly associated with the collapse were identified.

Upon arrival of emergency medical services (EMS), the patient was in cardiac arrest with asystole on electrocardiogram, dilated pupils, hypothermia, and jaw rigidity. The EMS team consisted of three emergency medical technicians trained in advanced life support. Manual cardiopulmonary resuscitation was initiated immediately, and the patient was transported to an ECPR-capable hospital.

On hospital arrival, rectal temperature was 14.0 °C. Rectal temperature measurement was used during the initial assessment because it was immediately available in the emergency setting before advanced temperature monitoring could be established.^10^ Arterial blood gas analysis obtained on hospital arrival revealed severe hyperkalemia, with a serum potassium concentration of 8.68 mmol/L.

Based on the environmental exposure and clinical presentation, accidental cold exposure hypothermia was presumed, and ECPR using veno-arterial extracorporeal membrane oxygenation (VA-ECMO) was initiated.^1^, ^2^ Cannulation in the emergency department was complicated by difficulty advancing the venous drainage cannula and persistent bleeding from the puncture site, which required careful hemostasis and secure vascular access. As a result, chest compressions were interrupted during the cannulation attempt. The total duration of interruption was approximately 20 min. This interruption was not intentional but was necessary to control bleeding and to safely establish extracorporeal circulation.

**Fig. 1:**
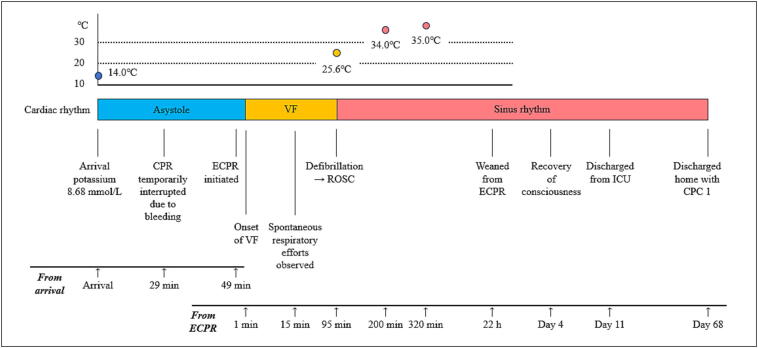
**Clinical course during resuscitation and rewarming**. The figure shows changes in core body temperature, cardiac rhythm, and key clinical events from hospital arrival to recovery. On arrival, the patient was in asystolic cardiac arrest with a rectal temperature of 14.0 °C and serum potassium of 8.68 mmol/L. Conventional cardiopulmonary resuscitation (CPR) was initiated, followed by extracorporeal cardiopulmonary resuscitation (ECPR) using veno-arterial extracorporeal membrane oxygenation (VA-ECMO). Chest compressions were temporarily interrupted during cannulation because of bleeding. Ventricular fibrillation (VF) occurred after ECPR initiation, and defibrillation at 25.6 °C resulted in return of spontaneous circulation (ROSC). The patient was weaned from ECPR after 22 h, regained consciousness on day 4, and was discharged home on day 68 with a favorable neurological outcome (CPC 1).

VA-ECMO was subsequently established in the angiography suite, and active rewarming was initiated immediately thereafter. Ventricular fibrillation was observed approximately one minute after initiation of VA-ECMO. Defibrillation was performed when the rectal temperature reached 25.6 °C, resulting in return of spontaneous circulation (Fig. 1).

VA-ECMO was discontinued 22 h after initiation. During the clinical course, pneumonia and pleural effusion were observed but did not progress to severe organ failure. The patient was managed in the intensive care unit for 11 days and underwent rehabilitation after stabilization. She regained consciousness on hospital day 4 and was discharged home approximately two months after admission.

Neurological outcome at hospital discharge was assessed as Cerebral Performance Category (CPC) 1.^11^ The patient returned to independent activities of daily living.

The predicted survival probability according to the HOPE (Hypothermia Outcome Prediction after Extracorporeal Life Support) score was 40%.^3^

## Discussion

The effectiveness of extracorporeal circulation for accidental hypothermia complicated by cardiac arrest has been well documented.^1^, ^2^, ^4^ Current guidelines, including those from the Wilderness Medical Society and the European Resuscitation Council (ERC) 2025 Special Circumstances guidelines, state that there is no lower temperature limit at which resuscitation should be withheld in hypothermic cardiac arrest.^5^, ^12^

To date, the lowest core body temperature reported in an adult survivor of accidental hypothermia was described in a case of immersion hypothermia, in which the blood temperature during afterdrop reached 13.7 °C; however, the rectal temperature on hospital arrival was 14.4 °C.^6^ In the present case, the rectal temperature on hospital arrival was 14.0 °C, representing one of the lowest reported arrival temperatures among salvaged adult patients.

Rectal temperature measurement was used during the initial emergency assessment because of its immediate availability. However, rectal temperature may overestimate true core temperature, particularly in profound hypothermia.^10^ Esophageal temperature measurement is recommended in intubated patients whenever feasible.^12^, ^13^

The favorable neurological outcome observed despite a suspected prolonged NFT is consistent with the well-established neuroprotective effects of hypothermia.^7^, ^14^ These principles are widely applied in both accidental hypothermia and therapeutic hypothermia, including deep hypothermic circulatory arrest during cardiac surgery.^15^

Serum potassium concentration has historically been used as a prognostic indicator in accidental hypothermia.^4^, ^16^ In the present case, the absence of trauma, asphyxia, or crush injury suggests that hyperkalemia was primarily related to hypothermia-induced cellular membrane dysfunction rather than irreversible tissue necrosis.^16^

The HOPE score predicted a survival probability of 40%.^3^ Importantly, no-flow time is not included in the HOPE score because it is often unknown in accidental hypothermic cardiac arrest, as most cases are unwitnessed or unmonitored.^3^

## Conclusion

This case demonstrates survival at one of the lowest core body temperatures reported on hospital arrival in an adult patient. It reinforces the established neuroprotective role of profound hypothermia and supports the use of ECPR in carefully selected patients with accidental hypothermic cardiac arrest.^1^, ^2^, ^5^

## Declaration of generative AI and AI-assisted technologies in the writing process

During the preparation of this manuscript, the authors used generative AI-assisted tools to support language editing and improve readability. The authors carefully reviewed and revised the content generated by these tools and take full responsibility for the integrity, originality, and accuracy of the manuscript.

## CRediT authorship contribution statement

**Hitoshi Kano:** Writing – original draft, Investigation, Conceptualization. **Masaki Nagama:** Investigation, Conceptualization. **Keisuke Bando:** Investigation. **Akio Endo:** Investigation. **Toru Takiguchi:** Writing – review & editing, Methodology. **Yutaka Igarashi:** Writing – review & editing. **Shoji Yokobori:** Writing – review & editing, Supervision.

## Ethics approval and consent to participate

This study was approved by the institutional review board of Sapporo City General Hospital (approval number: H29-055-377). Written informed consent for publication was obtained from the patient.

## Funding

The authors received no funding for this manuscript.

## Declaration of competing interest

The authors declare that they have no known competing financial interests or personal relationships that could have appeared to influence the work reported in this paper.
